# Self-management develops through doing of everyday activities—a longitudinal qualitative study of stroke survivors during two years post-stroke

**DOI:** 10.1186/s12883-016-0739-4

**Published:** 2016-11-15

**Authors:** Ton Satink, Staffan Josephsson, Jana Zajec, Edith H. C. Cup, Bert J. M. de Swart, Maria W. G. Nijhuis-van der Sanden

**Affiliations:** 1 Department of Occupational, Therapy and Research Group Neurorehabilitation, HAN University of Applied Sciences, Nijmegen, The Netherlands; 2 Department of Rehabilitation, Radboud University Medical Center, Radboud Institute for Health, Sciences, Scientific Institute for Quality of Health Care, Nijmegen, The Netherlands; 3 Division of Occupational Therapy, Department of Neurobiology, Caring Science and Society, Karolinska Institute, Stockholm, Sweden; 4 Department of Applied Social Sciences, NTNU, Norwegian University og Technology and Science, Trondheim, Norway; 5 Department of Rehabilitation, Radboud University Medical Center, Nijmegen, The Netherlands; 6 Research Group, Neurorehabilitation, HAN University of Applied Sciences, Nijmegen, The Netherlands

**Keywords:** Stroke, Self-management, Autonomy, Everyday activities, Participant observation, Longitudinal, Qualitative

## Abstract

**Background:**

A description of the complexity of the process of self-management and the way stroke survivors give meaning to their process of self-management post-stroke is lacking. This study explores how stroke survivors managed their lives, gave meaning to their self-management post-stroke and how this evolved over time.

**Methods:**

Data was generated through participant observations and interviews of 10 stroke survivors at their homes at 3, 6, 9, 15 and 21 months post-discharge. A constant comparative method was chosen to analyse the data.

**Results:**

*‘*Situated doing’ was central in stroke survivors’ simultaneous development of self-management and their sense of being in charge of everyday life post-stroke. Doing everyday activities provided the stroke survivors with an arena to explore, experience, evaluate, develop and adapt self-management and being in charge of everyday activities and daily life. The influence of stroke survivors’ partners on this development was sometimes experienced as empowering and at other times as constraining. Over time, the meaning of self-management and being in charge changed from the opinion that self-management was doing everything yourself towards self-managing and being in charge, if necessary, with the help of others. Moreover, the sense of self-management and being in charge differed among participants: it ranged from managing only at the level of everyday activities to full role management and experiencing a meaningful and valuable life post-stroke.

**Conclusions:**

The findings of this study indicate the doing of activities as an important arena in which to develop self-management and being in charge post-stroke. Stroke self-management programs could best be delivered in stroke survivors’ own environment and focus on not only stroke survivors but also their relatives. Furthermore, the focus of such interventions should be on not only the level of activities but also the existential level of self-management post-stroke.

## Background

Self-management is recognised as an increasingly important concept in stroke rehabilitation programmes [1–3]. Self-management is defined as an ‘individual’s ability, in conjunction with family, community, and the appropriate healthcare professionals, to manage the symptoms, treatment, physical, psychosocial, cultural, and spiritual consequences and inherent lifestyle changes required for living with a chronic disease’ [4](p.1145). Self-management encompasses dealing with the illness (medical management), but importantly also includes activating resources and living with a chronic illness in which emotional management and adjusting, meaning making and integrating illness into daily life (role management) are important processes [1, 5–8]. Stroke survivors described their self-management as a complex and long-term learning process during which they finally learned to live again with the of their next of kin [9]. Aside from individual self-management, stroke survivors stressed the importance of co-management with relatives [9]. To optimise the delivery and content of stroke self-management programmes, longitudinal studies are needed to further explore the complexity of the process of self-management post-stroke [1, 9].

Several longitudinal qualitative studies with stroke survivors reported how the process post-stroke involved restructuring and adapting physical, social and emotional aspects of an individual's life; social and emotional consequences were identified as the largest problems in daily life for stroke survivors during this process [10–12]. Stroke survivors’ return to daily life is complex; they see it as a challenging process of change with uncertainty and struggle to regain important roles and tasks in daily life [11, 13, 14]. Robison and colleagues [12] described after a one-year longitudinal qualitative study how stroke survivors had difficulties resuming valued activities post-stroke and how some stroke survivors were more adaptable than were others. Moreover, Kubina et al. [15] described in their longitudinal study how the process of stroke survivors’ re-engagement in valued activities over a two-year period was characterised by social connection and being in charge. In another longitudinal qualitative study with Norwegian female stroke survivors up to two years post-stroke, four phases were described: in the first phase participants’ main concerns were their bodily changes (0–2 months post-stroke); in the second phase they focussed on functioning in everyday activities (2–6 months); in the third phase most participants experienced a deeper self-understanding when they realised that the stroke represented a shift towards a new life (6–12 months) and in the fourth phase the participants were going on with their life, despite feelings of frailty and bodily ailments, which led to a decrease in activities (12–24 months) [16].

Although several findings of the aforementioned qualitative studies can be linked to stroke survivors’ self-management, none of them focussed specifically on the complex process of self-management post-stroke from a stroke survivors’ perspective. Furthermore, many longitudinal qualitative research projects studied stroke survivors up to one year after the stroke [10–12, 17–20], while the process of adaptation and self-management post-stroke often takes longer. Moreover, Robison [12] indicated that after only one year, stroke survivors are beginning to discover the extent to which they may or may not be able to resume valued activities; other authors have also recommended studying the process of self-management for stroke survivors beyond one year post-stroke [18, 20].

It appears that an explicit description of the complexity of the process of self-management and the way stroke survivors give meaning to their process of self-management up to two years post-stroke is lacking. To unravel the complexity of self-management in stroke survivors’ everyday life, we designed a prospective study with multiple points of data generation over a period up to two years post-discharge. This offers the possibility of analysing the process of self-management more in depth, as serial qualitative interviews offer considerable advantages over the more typical single ‘snapshot’ techniques in understanding patients’ changing experience of illness [21]. Moreover, we also wanted to collect observational data in addition to interviews. Participant observation could ground the study in daily life [22–24] and further unravel the complexity of self-management of stroke survivors in their own context. The present article draws on a research project that studied 10 stroke survivors up to two years post-discharge through participant observations and interviews. The current article presents the longitudinal qualitative study with the aims to explore how stroke survivors after discharge managed their lives, how they gave meaning to their self-management post-stroke and how this evolved over time.

## Methods

### Study design

To understand how stroke survivors manage their lives and give meaning to their self-management in the first two years post-discharge, a longitudinal qualitative study has been conducted by applying a constructivist paradigm [21]. The study was conducted between summer 2012 and winter 2014. The prolonged involvement in stroke survivors’ lives built trust with stroke survivors (and their context) and developed an accurate insiders’ understanding of the stroke survivors’ perceptions and experiences about their self-management of daily life after stroke in a narrative form [21, 25, 26]. The researchers followed ethical principles in accordance with Dutch research ethics regulations.

### Participants and context

In a period of 8 months, participants were consecutively recruited from stroke units from two rehabilitation centres. Potential participants received a letter with information about the aims and procedures of the study. Purposive sampling [22] was used, and participants were included who had experienced a first-time stroke, were living at home at least three months after discharge, were able to communicate in Dutch and had a score lower than 8 on the Hospital Anxiety and Depression Scale (HADS) [27, 28]. After the first five selected participants, we discussed within the research team which additional participants were needed in order to select a heterogeneous sample.

Twenty participants living in the east and south of the Netherlands have been approached for the study, and fourteen participants were interested in participating in the study. These participants have been visited by the first author and researcher (TS) and additional information was provided. Three participants did not meet the inclusion criteria: two participants had a HADS score higher than 8, and one participant had insufficient communication skills to participate in the interviews. One participant dropped out after the first moment of data generation. Ten participants participated in the study and represented a heterogeneous sample in terms of gender, living alone or with a partner, and left- and right-sided stroke (see Table 1). The participants received written and verbal information about the study and also gave their verbal and written informed consent to participate.

**Table 1:** Characteristics of participants

No. participant sex - year of birth	Diagnosis	Living conditions	Housing	Employment status	Leisure activities pre-stroke	(Dis)Abilities post-stroke after discharge
1 F - 1935	RCVA	Married No children	House Service flat, 1 year post-stroke	No paid job	Cycling, creating postcards, baking, physio fitness, reading, holidays	Walking with rollator outside house Decreased coordination left arm/hand—fatigue
2 M - 1958	LCVA	Married Children living away from home	House	Long-term disability	Doing odd jobs and woodwork in garden, computer, visiting antique markets with wife, walking	Walking without devices Decreased coordination right arm/hand—fatigue
3 F - 1942	RCVA	Widow Children living away from home	House Flat, 1½ year post-stroke	Retired pre-stroke	Playing cards, social activities with friends, activities of elderly association, aqua jogging	Walking with rollator outside house Decreased coordination left arm/hand—fatigue
4 M - 1946	RCVA	Married Children living away from home	Flat	Stopped working post-stroke	Visiting friends and family, attending soccer	Wheelchair inside house; mobility scooter outside Spastic left arm
5 F - 1946	RCVA	Living together with partner Children living away from home	Flat	Stopped working post-stroke	Creating postcards, playing badminton, playing saxophone, physio fitness	Walking with rollator outside house Decreased coordination left arm/hand Decreased concentration and attention—fatigue
6 M - 1951	RCVA	Living together with partner Children living away from home	Flat	Reintegrated in new job post-stroke	Visiting cultural activities, referee in rugby, position in board care institution, jogging	Wheelchair inside house; mobility scooter outside Walking few meters—paralysed left arm
7 F - 1948	LCVA	Widow Children living away from home	Service flat	Retired pre-stroke	Cycling, voluntary work (primary school)	Walking with rollator in and outside house Decreased concentration
8 F - 1946	LCVA	Married Children living away from home	Flat	Retired pre-stroke	Walking, visiting theatre and museums, tennis, baby-sitting grandchildren	Walking with rollator in and outside Wheelchair, mobility scooter and adapted bicycle outside Hemiparesis right arm—moderate fatigue Moderate attention in groups of people
9 F - 1957	RCVA	Widow Son and daughter living at home	House	Reintegrated post-stroke Stopped working 1 year post-stroke	Visiting friends, church, singing in choir	Walking, cycling and car driving without devices Moderate fatigue Decreased processing of visual and auditive stimuli
10 M - 1945	RCVA	Living together with partner Children living away from home	House	Retired pre-stroke	Gardening, horse riding, cycling, doing odd jobs	Walking with stick few meters in and outside Wheelchair and mobility scooter outside Hemiparesis left arm

*M* male, *F* female, *LCVA*, left cerebrovascular accident, *RCVA* right cerebrovascular accident

### Data generation

Researchers visited participants four to five times around 3, 6, 9, 15, and 21 months post-discharge. Most of the encounters started with a participant observation during a self-chosen everyday activity, followed by an interview. As we were aiming to explore how stroke survivors after discharge managed their lives in general and which possible factors could play a role in this regard, we explained that the participants could do the activities that were ´part of their daily life´ at that moment. The first and third author (TS & JZ) generated the data. Both were occupational therapists with experience observing persons with neurological conditions in daily activities at home and expertise in qualitative research.

The participant observations were mainly in or around participants’ homes but also at other locations, such as a workplace, health care centre, local park, supermarket, or a lunch room. A variety of daily activities and situations were observed (see Table 2). Some participants invited TS to join a therapy session, as this was for them an important activity. A few days before the visit, participants were phoned to remind them of the upcoming participant observation and interview. When conducting the participant observations, TS and JZ joined the participants in their self-chosen daily activities and situations. A specific observation protocol was not used. Within this open participant observation the researchers were ´part of the situation´ and followed the participants in the different situations. During most of the observations, keywords were written down about specific situations, solutions, problems or quotations to be used at subsequent interview.

**Table 2:** Diversity of observed activities and situations

Participant	Observed activities and situations
1	Baking a cake Doing the laundry Walking to the pharmacist and shopping Showing new apartment, preparing and drinking tea Walking in environment of new apartment Making postcards Preparing and drinking tea with participant and spouse
2	Preparing and drinking tea; woodworking in garden Joining occupational therapy session: exercises handwriting and woodwork Walking to garage and show repaired cars Walking in neighbourhood and drinking tea
3	Coffee, showing garden Preparing and having lunch at home Working in garden Preparing soup and having lunch at home Preparing and drinking coffee with pastry with participant
4	Drinking coffee with both spouses Showing pictures of jobs in past on computer Driving mobility scooter into park Visiting and training local soccer team Driving towards and shopping in builder’s store Showing new car with adaptations
5	Drinking tea with both spouses Making shopping list and shopping in supermarket Joining physiotherapy session Showing how to make postcards and making tea Touring in surroundings with mobility scooter Preparing and drinking tea with participant and spouse
6	Preparing and drinking coffee Using wheelchair to get to city centre and shopping Going to market with mobility scooter Showing adaptations and activities in kitchen Visiting and touring at new workplace Preparing and drinking tea ‘Walking and talking’ in environment in wheelchair
7	Preparing and drinking coffee Shopping in supermarket Having lunch in lunchroom in city Walking and talking, and showing car Performing little household activities, drinking tea with participant
8	Preparing and serving tea, coffee and cookies Folding laundry Preparing coffee and drinking coffee with participant and spouse Walking to garage and showing adapted bicycle
9	Visiting physiotherapy session Walking to and shopping in supermarket Preparing and having dinner with family Cycling in surroundings Driving with car to city Joining neuro-feedback therapy session
10	Preparing and drinking tea with participant and partner Showing garden and explaining activities pre-stroke Preparing and drinking tea with participant Trying out home trainer (cycle)

After the home visits, extensive field notes of the encounters with the participants were made on completing the participant observations [23, 24]. Sometimes the participants’ partners were involved in the activities. TS or JZ helped with small steps if this was requested or needed, which supported the process of building rapport [23]. Subsequent to the observations, researchers interviewed participants to elicit narrative material regarding the stroke-survivors’ self-management post-stroke. A set of general questions and topics were prepared for the interview (see Tables 3 and 4). The interview followed a conversational style [26]. Previous interviews with the participants and their observations shaped each subsequent interview. All interviews were tape-recorded and transcribed verbatim. Most encounters lasted between two and four hours. In total, 42 interviews and participant observations had been conducted during home visits as well as three telephone interviews instead of visits. Research assistants helped transcribe and organise the empirical data.

**Table 3:** Main topics for observations and interviews

• Self-management of participants • Performance of daily activities • Problem solving • Use of strategies • Roles	• Decision making • Support or help of others • Interaction with environment • Changes compared with previous point-of-data generation

**Table 4:** General Interview guide (adapted to moment of data collection on 3, 6, 9, 15, 21 months)

Introduction
Conversation with reflective questions about the activities the participants has done prior to the interview. After the conversation about the activities, the conversation continues with introductory, key, probing and reflective questions about self-management and related topics in different situation in the past 3, 6, 9, 15 and 21 months at home. - You are now at home for 3-6-9-15-21 months. What did you do in your everyday life in the past months? How did it go? How do you *manage the different situations in your everyday life*? How do you feel about that? Depending on the answer of the participants, probing question will be asked about: Self-management of participants, performance of daily activities, problem solving, use of strategies, roles, decision making, support or help of others, interaction with environment. - *Roles*: People often have roles, for example, in their family, social network or at work. A role is, for example, being a father, worker, partner, friend, etc. What kind of roles do you have at the moment? How do you feel about that? Depending on the answer of the participants, probing question will be asked about: self-management of participants, performance of daily activities, problem solving, use of strategies, decision making, support or help of others, interaction with environment. - *Interaction with and support of others*: Some people need others to manage a situation or they manage it themselves. How is that for you, are you managing yourself alone or together with people in your environment? How do you interact with the people around you? How do you feel about that? Depending on the answer of the participants, probing question will be asked about: self-management of participants, performance of daily activities, problem solving, use of strategies, roles, decision making. - *Changes*: You are now at home for 6-9-15-21 months. If you compare how you managed yourself 3–6 months ago with the manner in which you manage yourself currently, do you see any differences? How do you feel about that? Depending on the answer of the participants, probing question will be asked about: Self-management of participants, performance of daily activities, problem solving, use of strategies, roles, decision making, support or help of others, interaction with environment. - *Learning*: What did you learn about the way you can manage yourself in your everyday life? Depending on the answer of the participants, probing question will be asked about: self-management of participants, performance of daily activities, problem solving, use of strategies, roles, decision making, support or help of others, interaction with environment. - *Self-management:* o How do you manage yourself? Your everyday activities? Your life? o What helps you to manage yourself? Do you have ‘your own way’ (*strategies*) to manage yourself? o Are there examples of situations that limit you to manage yourself? o What have you learnt in the past months when you think about your own self-management? o Self-management often means that people make choices before or during they do something. How do you make your choices? Are there some changes in the way you make your choices? Are you taking these decisions yourself? o If you are asked to give advice to fellow stroke survivors, what kind of advice would you give them in regard to self-management? Depending on the answer of the participants, probing and reflective question about the different topics and possible changes. Summary of the conversation and ending questions: enable participants to reflect back on previous comments and make sure that nothing was overlooked. Closing of interview

### Data analysis

The method used for data analysis was informed by the constant comparative method [29, 30]. General analysis started after each encounter with participants to prepare for the next visit. In-depth data analysis began after all data was generated. The Atlas.ti software package (Atlas.ti Version 7.5.2) was used to assist in the process of data analysis. Initially, each separate case (all empirical data of one participant) had been analysed, followed by an analysis across all cases and finally a synthesis of the findings. During the close reading and comparison of the different categories, we paid extra attention to how participants reflected on their self-management to detect changes over time. Repeatedly emerging concepts were further analysed in their relation to self-management.

The use of memos and mind maps supported our analytical interpretation regarding participants’ meaning of their self-management post-discharge [30, 31]. During the entire analytical process, analysis and refinement of categories and themes was ongoing between TS, JZ and SJ (second author). Moreover, the analytical process and the preliminary and final themes were discussed and agreed upon in reflective meetings with all authors. For a description of the analytical process, see Table 5.

**Table 5:**
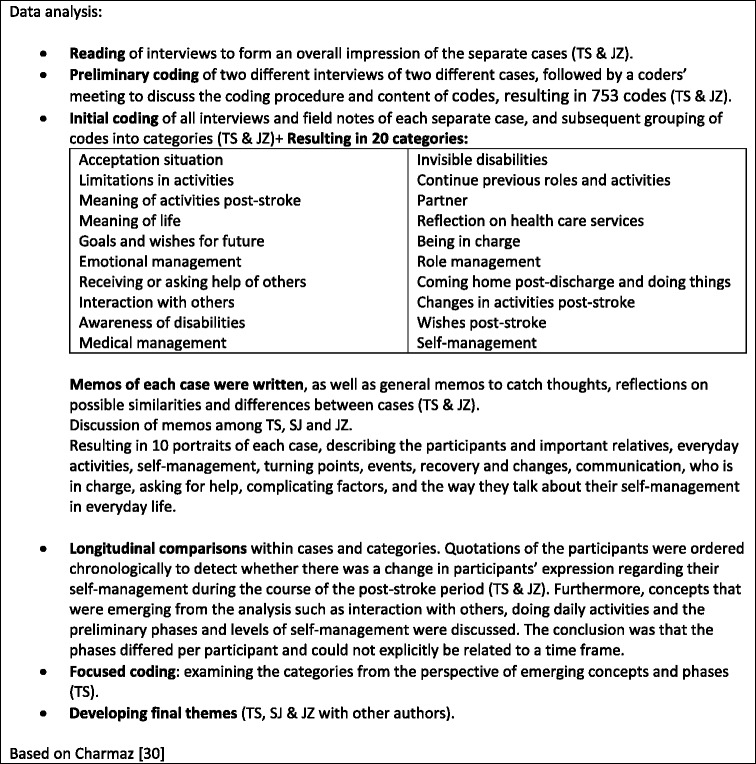
Analytical process

## Results

**Fig. 1:**
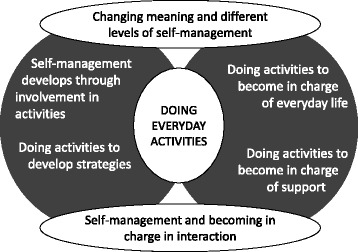
Doing everyday activities as a central element in self-management and becoming in charge

Six themes were developed in which the doing of everyday activities was a central element (see Fig. 1). To protect anonymity, quotes exemplifying the themes include the number of the participant and the moment of data generation (e.g., [3–4] means participant 3 in the fourth encounter).

### Self-management develops through involvement in activities

Self-management post-stroke developed gradually and was closely related to participants’ doing of everyday activities. However, doing activities was more than only performing an activity. Doing activities situated the participants in their everyday life, and through ‘doing’, participants interacted with their environment. ‘Doing’ encompassed exploration, adaptations, challenges and rewards (for example, when participants were able to fulfil pre-stoke roles), but doing also involved fear, saying farewell and grief (for example, when they had to decide that certain activities were not possible anymore).

While the participants were doing daily activities, different processes of self-management happened simultaneously, and through the ‘doing’, the participants experienced what self-management meant for them. ‘Doing’ provided participants with experiences that related to stroke—consequences, possibilities and disabilities in activities and situations, interaction with other persons, or receiving or asking for help. The ‘doing’ was the gateway to these experiences, and through these experiences most participants could learn. For example, one participant expressed: “*So that’s what you experience, and that’s what you have to deal with…that’s how it goes. Without facing it you can’t know it”* [7–2]. Not all participants reacted or learned from their experiences. During some participant observations, a few participants showed minor disabilities which they did not notice or correct, such as the participant [3–1] who did not use a new coffee pad in her single serve coffee machine whilst she was preparing coffee while at the same time talking to the researcher. During the interview, the participant trivialised this and said that she had not noticed this and that it was not a big problem for her because she could still manage to make coffee.

However, daily activities helped most participants understand how to manage their own situated doing: “*Within your familiar context you do things in a certain way, and then you realise that that method doesn’t work anymore. Not only because of physical limitations but also because of the context, the others around you, the practical aspects of the situation being different than what you had in mind. The context does not allow you to do what you had in mind”* [10–2]. Based on the experiences and appraisal of their ‘doing’, participants could decide to adjust their activity performance. One participant expressed: “*Just trying to do the things you used to do, and if it doesn’t work out, it doesn’t work out. Then you try to do it in a different way”* [4–5]. Through experimenting with alternative ways of performing activities, several participants experienced how they could still manage their daily activities but often differently than they did pre-stroke. One participant told how the housecleaning had changed over time post-stroke: “*I have become much more calm. First [pre-stroke-TS] I cleaned the silver or the copper very often, now I only polish it when it is dirty”* [3–3]*.* Through involvement in activities, participants learned how to self-manage, and this consequently supported participants’ sense of being a self-manager who could do everyday activities. This developed gradually, and through the ‘doing’ the participants experienced a sense of relative mastery. These experiences gave the participants access to further development, meaning that they could change and become the person who could manage disabilities, activities and, finally, self-manage their everyday life again.

### Changing meaning and different levels of self-management

With the ‘doing’ as a facilitator, the participants experienced and developed their self-management abilities in different ways. Over time, the meaning of self-management changed. Initially self-management connoted *“Independency and being able to practice it”* [6–1] and *“Try to manage everything yourself”* [2–2]*.* This transition was not easy for participants, many of whom felt lost post-discharge because they had missed coaching and support in self-management in their own environment directly after discharge.

The value attributed to independence in the context of self-management became less whilst the importance of meaning in choice attributed by participants in everyday situations increased. For example a participant said: “*Self-management gets more and more meaning. Before, self-management was planning…now I touch upon a deeper sense. Choosing between what comes on your path, does it feel right? What will it bring me? …in relation to my recovery to feeling better”* [9–3]. Finally, for most participants self-management meant that they could manage and direct their everyday life and do the things they wanted to do, like how one participant expressed that “*Self-management is about doing what matters to me. That I can do what I want and that that is okay”* [3–5]. Knowing what they were able to do post-stroke made most participants more confident in managing themselves; however, some participants discovered that that they could not do what they wished to do. During an observation, Participant 5 said she used to be able to manage several single activities, but that, eventually, she was not as active and could not do what she wanted as she used to do pre-stroke. She pointed to the Christmas tree in her living room and expressed that social activities around Christmas were fewer than what she did pre-stroke and that she was only sitting at home during the holiday. In the interview that followed, she said that she really missed having access to a person who could assist in finding new meaningful activities post-stroke.

Individuals varied regarding the scope and level of self-management. The sense of being a self-manager differed per participant and depended on several factors, such as participants’ abilities to self-manage, their perception of self-management, their personality pre-stroke and the interaction with their environment. Some participants talked about self-management as self-managing on the level of activities. Other participants perceived it more broadly, as they strived to be engaged in a meaningful life and meaningful roles. One participant who, despite her severe stroke, was able to self-manage together with her partner and focused more on managing and directing her own life said: “*Self-management is taking charge of your own life”* [8–4]. Through the ‘doing’ she became a self-manager and felt empowered to direct her own life. Moreover, the participants who expressed a strong sense of self-management showed during observations that they interacted fairly well with their environment. They dared to ask for help and arranged facilities to optimise their mobility. Furthermore, they dared to decide not to do certain activities, even when it was still possible, as participant 7 expressed about her administration. During an observation, she presented letters from several authorities and reported that she had asked her daughter-in-law to do her administration. This participant perceived herself as a good self-manager and said that deciding not to do something any longer was also part of that.

### Doing activities to develop strategies

‘Doing’ was also essential to develop strategies. Through the ‘doing’, the participants became aware of their limitations post-stroke and possibilities to manage them. Several participants stressed that doing activities at home was needed to experience and learn strategies, like the woman who reflected on exercises and strategy training in the rehabilitation centre and explained that she had to experience it herself: “*Because my whole life is turned upside down… So how can I answer how I am going to approach it. I don’t have a clue; I have to experience it all, in order to know what works or doesn’t work”* [7–2]. Although individuals varied in the type and timing of strategies, medical, role and emotional management strategies were mostly interwoven. However, in the first post-discharge period, self-management strategies were more related to medical management of the stroke consequences. The most often-heard strategies involved pacing to manage fatigue and using a shopping list and agenda to compensate for the minor memory problems. Several participants experienced invisible problems. One participants explained: “*I experience regularly that I suffer from ‘fatigue in my head’, too much tingling, and eh, it feel as everything is tickling, entering hard and loud, so that is difficult*” [9–2]. Regarding ‘invisible problems,’ such as concentration loss or hypersensitivity for sensory stimuli, the ‘doing’ also provided the participants with experiences and consequently with a better understanding of the real stroke consequences and possible strategies to cope. Remarkably, several participants who experienced invisible problems did not explicitly talk about them during the first encounters. It seemed that it took some time before the participants became aware of and understood their invisible problems. Moreover, only one participant had received additional coaching to manage the invisible problems. None of the others had received professional coaching but had just learned to manage their invisible problems by trial and error in daily life.

Gradually, the understanding about their stroke consequences and their strategies to cope increased. In the first interviews post-discharge, several participants expressed how their limitations were not clear and sometimes just happened to them. During the last interviews, the participants expressed that they had learned which strategies worked for them to compensate for the limitations. Instead of being surprised and reactive, most participants became proactive and were able to manage themselves and their activities: “*So when I go see my family, I ask myself the question: what is the easiest and safest way to get there?*” [4–3]. Many participants mentally planned the activity before the actual ‘doing’. Although most participants learned step by step to self-manage their everyday activities, there were also situations where participants were not successful in self-managing. An example of this occurred when a researcher arrived for a visit at a participant’s home and the participant was in panic [9–5]. At the same time the researcher arrived, her new cat had knocked over several objects in the kitchen and the phone rang. The participant needed the researcher’s help to calm down and get a grip on the situation again. Later on in the interview, the participant explained that post-stroke it took her more time to solve problems, to self-manage these situations and to relax afterwards. She expressed: “*When everything comes together, like the cat who made a mess in the kitchen, my daughter with a difficult question on the phone, and just being tired… then it is not easy. I need more time in those situations to understand what to do”* [9–5].

After participants had learned strategies to self-manage the stroke-consequences, the participants gradually related their strategies to the performance of more demanding activities. One participant expressed how he cooked again for his wife and friends, but the recipes and ingredients had changed: “*On a regular basis I take out something ready-made … typically stew, stir-fried or vegetable dishes, so always meals you can prepare with one hand”* [6–3]. After a while the strategies became part of participants’ routines in daily life. Eventually, all participants related their self-management strategies to activities of daily life. Self-management strategies were embedded in and got meaning out of everyday activities. Participants rested before going to the theatre; they asked for help in a shop to buy their groceries for cooking or they switched off their emotions to be present at a birthday party. Although the strategies eventually helped the participants manage and do their everyday activities, several participants still felt that the strategies were insufficient to fulfil all valued roles as they did pre-stroke. Participant 8, for example, expressed sadly how she could not be the grandmother she was before the stroke: “*If I think for example about the things I cannot do anymore with my grandchildren, that makes me sad. Yes, as a grandmother you wish to be a nice grandmother, and I think I was. If they went swinging, grandmother joint them. … Well, I cannot do that anymore and that makes me sad”* [8–2]*.*

Some strategies took on other meanings over time, especially that of taking rest. Initially, rest was perceived as an extra activity performed before or after a daily activity; participants had to get used to it. In later interviews, participants’ resting had become integrated in their daily routines. Rest now had a positive meaning; one participant expressed: *“It happens on occasions that I am too tired, and I am just lying down with a book on the sofa. When I do that because I am too tired to do something, I enjoy the rest”* [1–5].

### Doing activities to become in charge of everyday life

When the participants talked about self-management, many simultaneously talked about their sense of becoming in charge. One participant stressed in her last interview “*Self-management … it is all about directing your own life*” [7–4]. Participants became in charge in different areas. First, by using strategies, the participants became in charge of their stroke consequences during their ‘doing’. Subsequently, the ‘doing’ supported participants’ sense of being in charge of their daily activities, like a participant expressed in relation to his self-care: *“A fundamental transition was regaining the ability to choose myself when I go to the restroom because it became physically possible again”* [6–1]. The experience of mastery of activities was satisfying for most participants: “*For example, doing stuff in the house that requires some efforts, e.g., sorting out clothes … that gives me satisfaction. The ability to do things for myself and by myself*” [8–2]. Moreover, the sense of being in charge of activities also gave the participants the confidence to create, manage and become in charge of other new and engaging activities. A participant who had woodwork as a hobby said: “*I appreciate discovering something new again, like new tools, how do you use them … what is the mechanism behind them, how do you make it, how can you improve it?*” [2–2].

Another way participants became in charge of activities was doing activities earlier than their partner did them. They related this to attaining ownership of single activities through ‘doing’. When they did the daily activity before, it could have been taken over by their partners; it gave them a feeling of ownership and sense of being in charge of the activity again. One participant said: “*The last thing I reclaimed was the coffee machine … and I have done that all by myself. When she comes home from her work coffee is ready, and that … she can’t take that away”* [2–3].

With regard to becoming in charge and directing their lives, most participants expressed that they gradually had become the ones who decided what, when and how they could do something. However, for some participants, being in charge was easier said than done, such as a participant who explained: “*In the morning I am full of energy and do a lot of things. In the afternoon I am just too tired to do things*” [6–4]. Being in charge of single activities was one thing, but being in charge and finding meaning in post-stroke everyday life was something else. Several participants had expressed in interviews how their “*life was ending*” [2–2] or had lost meaning and was “*just babbling on*” [6–2], although participant observations had showed how they self-managed and were in charge of single activities independently.

Regarding the sense of being in charge and directing their lives, mobility was a specific aspect. Mobility was not only the ability to walk, but also, for example, the ability to drive a mobility scooter or a car. Increasing mobility gave the participants possibilities to broaden their world and to decide and feel in charge of when and where to go. The ability to go outdoors independently was for some participants related to their physical recovery, and for others also to the use of resources and arranging mobility scooters, adequate wheelchairs or adapted cars. One participant expressed how the mobility scooter had empowered her to expand her social life again: “*In the end you do meet new people again. Slowly but surely your little world expands, and I am proud to say out loud: that’s my own merit*” [7–4]. Through increasing mobility, the participants could more easily decide themselves when and where to go, and this gave the participants a sense of being in charge of their social life. To the contrary, not all participants could easily arrange assistive devices such as a mobility scooter or an adapted car. Often participants needed good interactive skills and an understanding of their insurance or municipal regulations, which was not easy for every participant.

### Doing activities to become in charge of support

Receiving support as part of self-management was a topic that came back in all encounters. How much support was needed was only determined in the actual ‘doing’. In the first period post-discharge, several participants perceived the help of others as necessary. Although most participants felt uncomfortable receiving or asking for help, they realised that without the help of others, they could not manage and complete an activity or go outside their house. However, over time, participants’ attitudes towards the support of others in relation to their own self-management changed.

Gradually, the support of others was perceived differently by the participants: from help which was needed in the beginning post-discharge towards help which was not necessary or sometimes even unwanted. Moreover, through their ‘doing’ and interaction with others, the participants developed another sense of being in charge of support, compared with the feeling they had just after their stroke. In the last interviews, several participants who still needed certain support expressed that asking for help still felt uncomfortable, but that they now perceived themselves as the ones who were asking for help or who could even instruct their partners how to do activities. Like one participant reflected: “*Of course I’d prefer to do everything myself, but when I can’t, then I find it easy to delegate it and to give somebody else the instructions how to do it*” [1–5]. For most participants, asking for help or getting help no longer denoted dependency on the other person, but asking for help was part of their self-management, like another participant expressed: “*Asking for help is a learning process. I decide when and how much help I receive. Support is part of the game”* [6–4]. They asked by choice and not as a result of need.

### Self-management and becoming in charge in interaction

In and through their ‘doing’, participants interacted with other persons and developed ways to self-manage or to co-manage with their partners. Most couples needed time to share experiences and discuss how to co-manage certain situations together. One participant expressed: “*It is about having a dialogue about it, I mean, I just can’t decide on my own to do one thing or another. And when she [partner—TS] wants to do something, then share what the plans are. I believe we both found a good balance between us again*” [6–4]. In most cases, both partners were involved in co-managing daily life and out of reciprocity they also considered how to take care of each other. It was a give-and-take, and often the balance was experienced as positive and supportive. However, a few participants explained how they did not always feel supported to self-manage and become in charge. They related this often to their partner who helped too quickly. In most cases, the spouses could discuss this, like a participant expressed: “*I tell him: ‘I want you to give me more space to do things myself. Instead of encouraging me to do something, you are telling me that you will do it… and I want to do it myself’”* [1–2]. On the other hand, some participants felt less empowered to self-manage and be in charge of daily activities by their partners. In the last interview, participant 5 expressed that she did not do so much at home, but in a joint interview, her husband expressed that he had taken over the household and shopping because that was his way to self-manage. There was not much interaction about each other’s experiences, and the participant said that they had stopped talking about it.

Regarding the interaction in relation to self-management, several participants expressed that communication was an important skill in self-management, especially communication in relation to arranging resources and asking for help. One participant expressed that “*If you have difficulties to ask for help, then self-management will get difficult*” [6–3].

## Discussion

The current findings support the individual, dynamic and contextual nature of self-management [7–9, 32, 33]. However, this research adds to this knowledge how participants’ self-management and sense of being in charge were interwoven, and developed and attained meaning through ‘doing’. For stroke survivors the value of doing activities is described in several other publications [12, 15, 16, 19, 34, 35], but the current findings show how doing everyday activities provided the participants with experiences on different levels in relation to their sense of self-management and being in charge. Through everyday activities the participants experienced their stroke consequences, and, subsequently, the participants learned to self-manage the stroke consequences in light of everyday life. This ‘feedback from doing’ [35] helped participants to explore (new ways of) everyday doing, adapt their performance and expectations regarding their everyday doing and re-engage in valued everyday activities. An implication from the findings is that professionals in stroke rehabilitation should use the value of doing everyday activities [36] in stroke self-management programmes to create an arena where stroke survivors can self-manage and become in charge of the stroke consequences and daily activities on a practical level.

Several participants used strategies proactively to self-manage stroke consequences when preparing and exploring new or challenging activities. This finding supports the assumption of Tielemans and colleagues [37] about the value of proactive action planning in self-management programs. The current study adds to this assumption that our participants related their proactive planning to meaningful activities. This suggests to rehabilitation professionals that the delivery of self-management programs should be situated in meaningful context. In addition to information provision, goal setting and action planning, pro-active self-management strategies should be learned, applied and evaluated in the context of the everyday life of stroke survivors to do justice to the complex nature of self-management.

Another finding of the current study is how the meaning of self-management and being in charge changed over time. Initially, our participants connected self-management with doing everything themselves, if possible, without any help. Eventually they experienced asking for help not as a result of need, but as a result of choice and felt in charge. Regarding the sense of being in charge, our findings support the study of Kubina et al. [15], who described how ‘doing’ supported participants’ sense of personal agency [38] in daily activities and meaningful life. However, Kubina et al. [15] presented being in charge only as considering oneself a primary decision maker regarding how and when to resume personally valued activities. In this study, we found that being in charge was also about being in charge of stroke consequences, one’s own life and requesting the help of others.

As in other studies [9, 12, 14, 39], the current findings revealed the influence of the partner on participants’ ability to resume pre-stroke activities, self-management and their sense of becoming in charge. Our findings showed on one side how partners facilitated the self-management process, but on the other side how they sometimes were a barrier for stroke survivors’ self-management by taking over everyday activities too quickly. Furthermore, being in charge didn’t mean that the participants were independent and totally self-managing, but often that they were interdependent and co-managing with their partners in various situations which changed over time [39, 40]. Interaction with others in the network of participants, exchanging experiences, and asking and receiving support were important elements. This suggests that rehabilitation professionals should include the relatives of stroke survivors as soon as possible in the stroke self-management programs. This will assist in addressing topics such as being in charge, interdependency, the value of supporting the stroke survivor, asking for help, and co-management in relation to participants’ empowerment.

Concerning the scope of self-management and being in charge, there were differences among the participants. The findings of this study revealed that creating meaning in life after stroke was not easy for every participant. Although several participants felt able to self-manage a new life with meaningful activities post-stroke in the second year post-discharge, this was not experienced by all participants. Some participants only experienced ‘self-managing the practicalities of life’, but not on an existential level of living with a stroke and creating meaning in life. The different levels of self-management post-stroke reflect the findings of a focus group study among allied health professionals about stroke self-management [41]. However, the current findings emphasise that becoming a self-manager at different levels from single activities to the existential level takes time [7, 16]. It appears therefore that in addition to post-discharge self-management programs that focus on practical issues in everyday life, longer term programs that facilitate self-management on a more existential level are needed. These should focus on role management rather than the illness or impairment (medical management), and should start once the stroke survivor has spent a considerable number of months at home, for example twelve months post-discharge. The timeframe will vary depending on the state of readiness of the stroke survivor and the nature of their questions about their roles post-stroke [5, 7, 8, 42]. Moreover, the doing of everyday activities as part of a defined role, can help stroke survivors learn from experiences, recognise oneself, define (new) meaning in their ‘doing’ [12, 16, 19] and address the existential dimension of self-management.

### Methodological considerations

The number of participants is limited and restricts the generalisability. Although diversity was achieved in gender, living conditions and disabilities, the sample does not represent the general population of stroke survivors. Participants with severe cognitive impairments and severe communication problems, or participants who were dependent on others for their mobility, were not represented. However, the findings of this study reveal how a certain group of stroke survivors self-managed and gave meaning to their self-management post-stroke. This can be applied to other stroke survivors or contexts. One strength of this study was the extended period of data generation which provided the stroke survivors with the possibility to reflect on their own process of self-management post-stroke [21]. Although a constructivist paradigm assumes that knowledge is created between the participants and researcher on the moment of data generation [21], we have followed different strategies to enhance the trustworthiness of the findings: triangulation of data collection methods (participant observations and interviews) and triangulation of researchers (use of two researchers for the data collection and the analysis (see Table 5)). This has enhanced the completeness and credibility of the findings. However, some participants may have altered their behaviour because they were being observed. A few participants initially expected that the observations would be an assessment of problems and disabilities, but the researcher explained that the intention was to ‘be part of their daily life’ to observe how they self-managed, and, for example, asked for help as part of their self-management instead of focussing on disabilities or independence. Another strength of the study was that its design grounded it in the everyday life of stroke survivors. This might have influenced our findings, as the participant observation during the doing of activities was part of the method and, therefore received extra attention. However, during interviews, the participants, without being prom expressed in various ways the value of their everyday doing in relation to self-management and the process of becoming in charge. The self-chosen activities can be considered as a strength. The possibility to choose the activities fits with the client centred approach and the attempt to support the participants in being in charge during the moments of data generation, which also fits the assumptions of self-management interventions. However, the participants might also have chosen activities that caused not too many problems, which might not have given the full picture of self-management in different situations.

On occasions where partners were present, data generation was adapted to include the partner in the interviews or observations and to capture the interaction between the stroke survivor and the partner. Furthermore, reflective meetings with all authors to discuss the analytical process and the preliminary and final themes enhanced the credibility of the study [43, 44].

## Conclusion

This study aimed to explore stroke survivors’ self-management post-discharge. The findings indicated that situated doing was central in stroke survivors’ simultaneous development of self-management and the sense of being in charge of everyday life. This process was individual, dynamic and contextual, in which the interaction with the partner was experienced empowering in some situations and as a constraining factor in others. It did not appear possible to describe a general scenario for the development of self-management and becoming in charge of everyday life. Experienced levels differed among participants, ranging from only managing at the level of everyday activities to full role management and experiencing a meaningful and valuable life post-stroke. Individualised self-management programs for stroke survivors, together with their relatives, should be offered post-discharge in stroke survivors’ homes to coach stroke survivors and their relatives in the development of self- and co-managing with the doing of everyday activities as an essential determining part of everyday life. Moreover, stroke self-management programmes with more existential content should also be considered after about one year of living at home to address the need of stroke survivors to live a meaningful life post-stroke.
